# Development of an electrooculogram-based eye-computer interface for communication of individuals with amyotrophic lateral sclerosis

**DOI:** 10.1186/s12984-017-0303-5

**Published:** 2017-09-08

**Authors:** Won-Du Chang, Ho-Seung Cha, Do Yeon Kim, Seung Hyun Kim, Chang-Hwan Im

**Affiliations:** 1 School of Electronic and Biomedical Engineering, Tongmyong University, Busan, Republic of Korea; 2 Department of Biomedical Engineering, Hanyang University, 222 Wangsimni-ro, Seongdong-gu, 04763 Seoul Republic of Korea; 3 Department of Neurology, College of Medicine, Hanyang University Hospital, Seoul, Republic of Korea

**Keywords:** Electrooculogram (EOG), Saccade, Eye-writing, Amyotrophic lateral sclerosis (ALS), Human-computer interface (HCI)

## Abstract

**Background:**

Electrooculogram (EOG) can be used to continuously track eye movements and can thus be considered as an alternative to conventional camera-based eye trackers. Although many EOG-based eye tracking systems have been studied with the ultimate goal of providing a new way of communication for individuals with amyotrophic lateral sclerosis (ALS), most of them were tested with healthy people only. In this paper, we investigated the feasibility of EOG-based eye-writing as a new mode of communication for individuals with ALS.

**Methods:**

We developed an EOG-based eye-writing system and tested this system with 18 healthy participants and three participants with ALS. We also applied a new method for removing crosstalk between horizontal and vertical EOG components. All study participants were asked to eye-write specially designed patterns of 10 Arabic numbers three times after a short practice session.

**Results:**

Our system achieved a mean recognition rates of 95.93% for healthy participants and showed recognition rates of 95.00%, 66.67%, and 93.33% for the three participants with ALS. The low recognition rates in one of the participants with ALS was mainly due to miswritten letters, the number of which decreased as the experiment proceeded.

**Conclusion:**

Our proposed eye-writing system is a feasible human-computer interface (HCI) tool for enabling practical communication of individuals with ALS.

## Background

Individuals suffering from amyotrophic lateral sclerosis (ALS) gradually lose their ability to control muscles. Since eye movement is one of the few communication methods available until the later stages of this disease [1, 2], many researchers have attempted to devise communication tools utilizing this remaining eye function. If parents or guardians of individuals with ALS can recognize slight eyeball movements or subtle eyelid blinks, they can communicate with these individuals using specially designed alphabet boards or communication charts [3]. Recent developments of assistive technologies utilizing eye tracking allow patients with ALS to express themselves independently, thereby significantly enhancing their quality of life [4–6].

Two different methods have been developed for automatic eye movement tracking: 1) eyeball tracking utilizing optical or infrared cameras and 2) eye tracking using electrooculogram (EOG). Camera-based methods have shown to achieve higher angular precision than EOG-based methods [7]. In practice, a number of camera-based eye-trackers are commercially available (e.g., iAble**,** DynaVox, EyeMax). However, this method is subject to a number of limitations, including relatively expensive price, difficulties in system setups, and inconsistent recognition rates due to the variability of eyelid/eyelash movements between individuals and differences in ambient brightness level [1, 7–9]. EOG-based methods can be used as alternatives to camera-based methods because most of the limitations of the camera-based methods do not apply to the EOG-based methods. For example, EOG-based eye tracking devices are more economical to manufacture and are not influenced by lighting or the physical conditions of the eyes. However, the applications of EOG-based methods are limited by their low spatial resolution. For instance, it is generally difficult to estimate an absolute gaze position from EOG signals because of signals from other sources in the human body [10]. For this reason, most EOG-based applications had been able to classify up to only eight saccadic eye movement directions [11–15] until Yan et al. classified 24 eye movement directions with a wide screen (150° horizontal visual angle) and a fuzzy system [16]. Using Yan et al.’s system, the mean accuracy of three subjects was reported to be 87.1% [16]. Nevertheless, the performance of this system was not stable; gazes were often misclassified as adjacent positions, showing accuracies lower than 80% at almost half of the positions. Moreover, turning eyes 75^o^ to the left/right from the center may be difficult even for healthy users, making it even more problematic for individuals with ALS [9].

Recently, a new type of EOG-based method was introduced to overcome the main limitations of classical EOG-based methods. Specifically, Tsai et al. proposed a system for writing Arabic numbers by drawing number shapes using eye gaze in a process termed *eye-writing* [17]*.* This process was developed with the aim of aiding communication in individuals with ALS, but was evaluated with healthy participants only. Recently, the *eye-writing* concept was applied to the English alphabet [18], where *eye-writing* could significantly increase the number of choices for a given individual. The mean F1 score for classifying 36 patterns was reported to be 87.38%, even without any individual optimization.

Although these results are encouraging, the question of whether *eye-writing* is feasible for individuals with ALS remains unanswered. Most of the previous studies on EOG-based eye tracking were conducted only on healthy participants. Although Kaethner et al. reported results from a subject with ALS, the binary classification rates varied from 60 to 100% over different trials [9]. It is important to perform feasibility tests with individuals with ALS for the following two reasons: First, the saccadic patterns of individuals with ALS may differ from those of healthy people. For instance, decreased saccadic velocities were often (4 out of 9) observed even in the earlier stages of ALS due to the oculomotor deficits that are common in ALS [19]. Second, it may be difficult for some individuals with ALS to learn how to *eye-write* specific patterns. Since drawing patterns with the eyes is an unusual behavior even for healthy people, we found that preliminary training sessions demanding immediate responses from the user were always necessary [18]. However, some individuals with severe ALS might have difficulty expressing whether they are accustomed to the *eye-writing* task.

In this paper, we propose a new *eye-writing* system for individuals with ALS. This system includes 1) a series of computational algorithms to reconstruct eye movement traces and to more accurately recognize 10 Arabic numbers, 2) new designs of Arabic numbers aimed at facilitating *eye-writing*, and 3) a training procedure to efficiently explain to the user, with minimal user feedback, how to *eye-write*. We validated our proposed system with healthy participants and individuals with ALS.

## Methods

### Participants

A total of 23 participants (20 healthy participants and three individuals with ALS) were recruited for this study. Prior to the experiments, all participants received a detailed explanation about the research purpose and design and provided written consent. The study protocol was approved by the Institutional Review Board (IRB) of Hanyang University Hospital. Among the 20 healthy participants (15 males and 5 females, mean age 24.2 ± 4.17 years), data from two were discarded due to severe artifacts caused by sweat. Six of the 18 healthy participants wore glasses/lenses. The first individual with ALS (female) had been diagnosed for 4 years, aged 59 years, and the ALSFRS (ALS functional rating scale) was 17 at the time of the participation. The second individual with ALS (male) was aged 63 years, had been diagnosed for 3.5 years, and the ALSFRS was 18. The third individual with ALS (male) had been diagnosed for 8 years, aged 41 years, and the ALSFRS was 25. The ALSFRS is a well-known measure for evaluating the functional status of individuals with ALS and is based on a questionnaire. The questionnaire evaluates daily activities in 12 categories: speech, salivation, swallowing, handwriting, cutting food, dressing and hygiene, turning in bed, walking, climbing stairs, dyspnea, orthopnea, and respiratory insufficiency [20]. The average ALSFRS score for a healthy participant is 48, while the score of a patient with ALS in a completely locked-in state is 0. Throughout the experiments, caregivers helped the experimenters to communicate with the participants with ALS.

### Experimental environments

EOG signals were acquired using an ActiveTwo biosignal recording system (Biosemi, Amsterdam, Netherlands) at a sampling rate of 2048 Hz. Four electrodes were placed around the eyes: two on the left and right sides of the eyes, and two above and below the right eye. A common mode sense electrode and a driven right leg electrode, which functioned as a reference and a ground electrode, respectively, were placed at the left and right mastoid. Prior to electrode attachment, the skin was cleaned with antiseptic wipes to eliminate sweat or other materials that could interfere with signal acquisition.

**Fig. 1:**
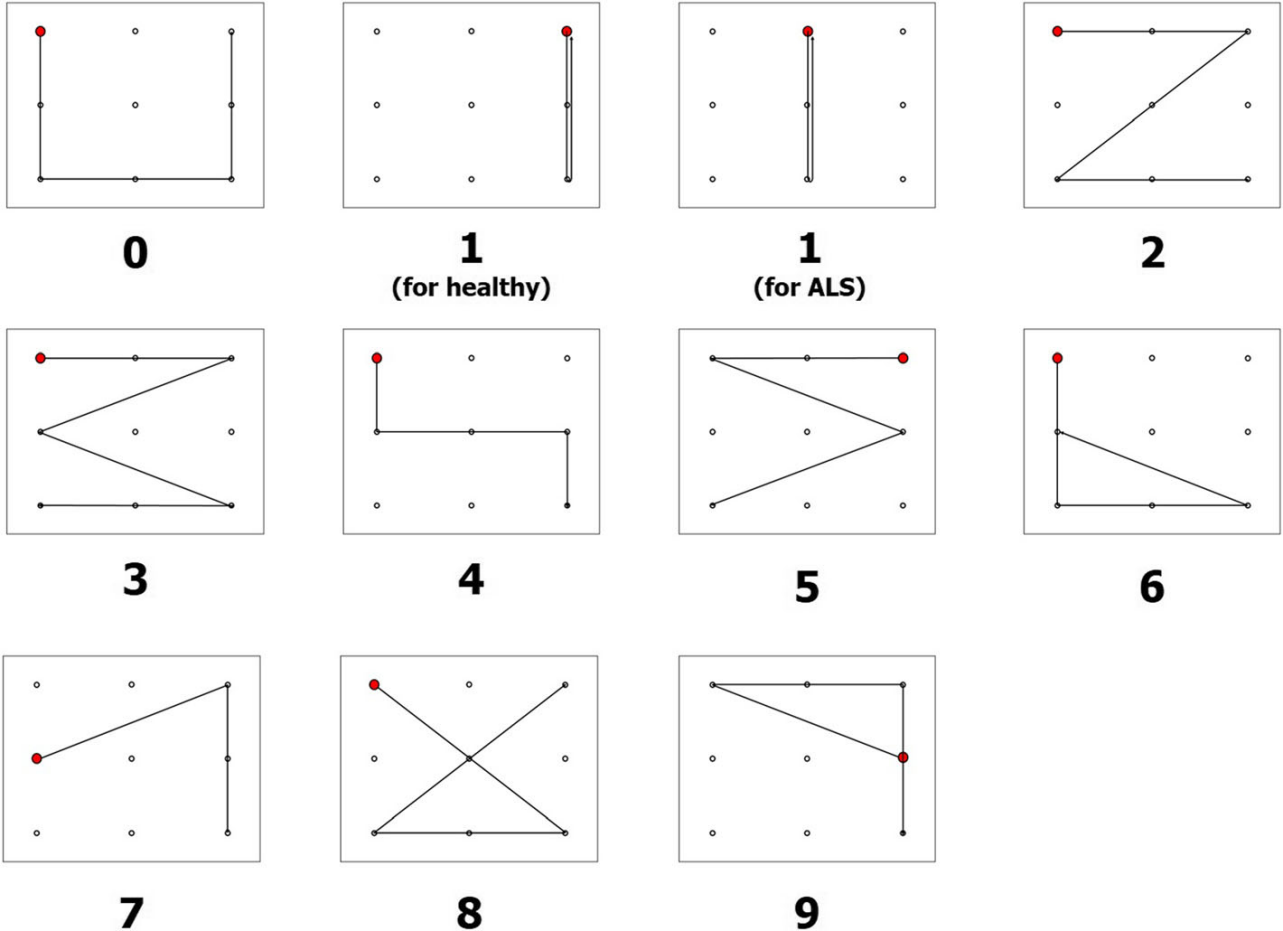
Pattern designs of the Arabic numbers

We designed 10 different *eye-writing* patterns, each corresponding to an Arabic number. These patterns were designed to minimize eye movement but to maintain the shapes of the original numbers (see Fig. 1). A red dot in each pattern indicates the starting point of *eye-writing*, and an arrow indicates the end point. The same patterns were used for healthy participants and participants with ALS; however, the pattern of the number ‘1’ was shifted to the center of the canvas to facilitate *eye-writing* by participants with ALS.

Data acquisition was controlled using E-Prime software (Psychology Software Tools, Inc., Sharpsburg, PA, USA). This software was also used to display numbers or other graphical instructions on a monitor with auditory instruction. All the raw EOG data recorded from healthy participants during the experiments are available at the EyeWriting repository (https://github.com/EyeWriting/EyewritingNumber), but data from participants with ALS are not shared because IRB did not allow sharing of this dataset.

All experiments with healthy participants were conducted in a quiet room. Each participant was asked to sit on a comfortable armchair in front of a 24-in. monitor. The width and height of the monitor were 61 cm and 28.5 cm, respectively. Each participant was asked to place his/her head on a chin rest in order to minimize head movement, and the height of the chin rest was adjusted according to the participant’s preference. The distance between the monitor and the participant was set to approximately 62.5 cm, making 52.02° maximum visual angle that participants could move their eyes horizontally.

The experiments with participants with ALS used a different display device. Specifically, the first two experiments with participants with ALS (No. 19 and No. 20) were conducted in a hospital room, where the visual stimuli were projected on a wall using a video projector. The size of the projected screen was approximately 135 cm (width) × 102 cm (height). For the third experiment (participant No. 21), a 55-in. [86 cm (width) × 48 cm (height)] television was installed in the participant’s home. During the experiment, the participants were seated either in a wheelchair approximately 150 cm away from the projector screen (participants 19 and 20) or 125 cm away from the TV display (participant 21). Of note, a chin rest was not used for the participants with ALS.

### Experimental procedure

**Fig. 2:**
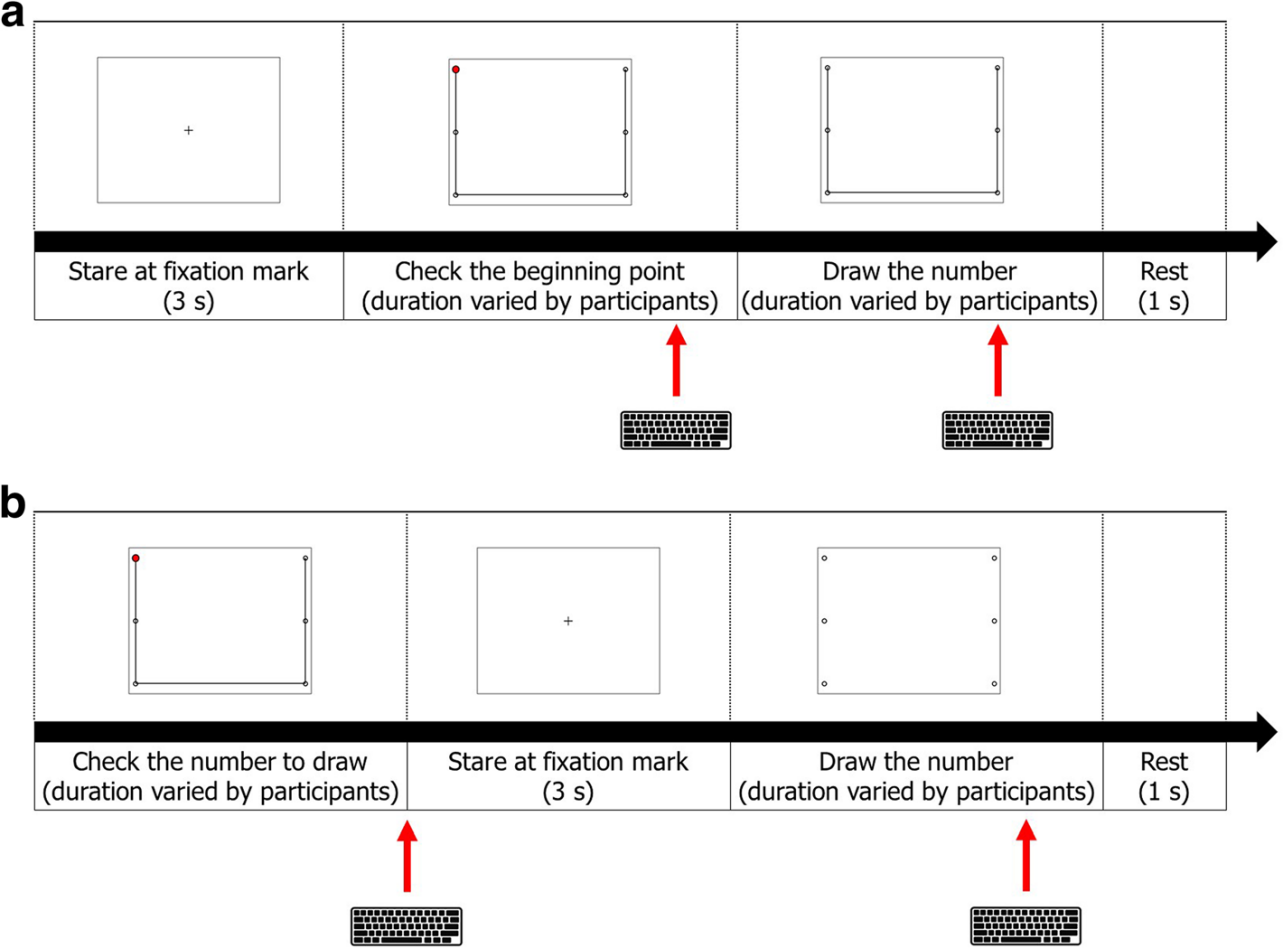
Experimental procedures of the two sessions for healthy participants: **a** practice session, **b** exercise session. The keyboards indicate that each slide finishes with input from the keyboard

The experimental procedure for healthy participants consisted of two consecutive sessions: a practice session and an exercise session (Fig. 2). During the practice session, the participants were asked to *eye-write* the number patterns along the given guide lines; the guide lines were not provided in the exercise session. For both sessions, the participants were instructed to *eye-write* each number in three steps. In the first step, each participant fixed his/her gaze at a fixation mark for 3 s. After keeping the shape of the number in mind, the participant began to *eye-write* the number on the 3 × 2 grid. The practice session was repeated until each participant became accustomed to *eye-writing*, without recording any EOG signals. The exercise session was then repeated three times; EOG signals were recorded during these sessions. The time durations for *eye-writing* a number varied every time the participants *eye-wrote*.

**Fig. 3:**
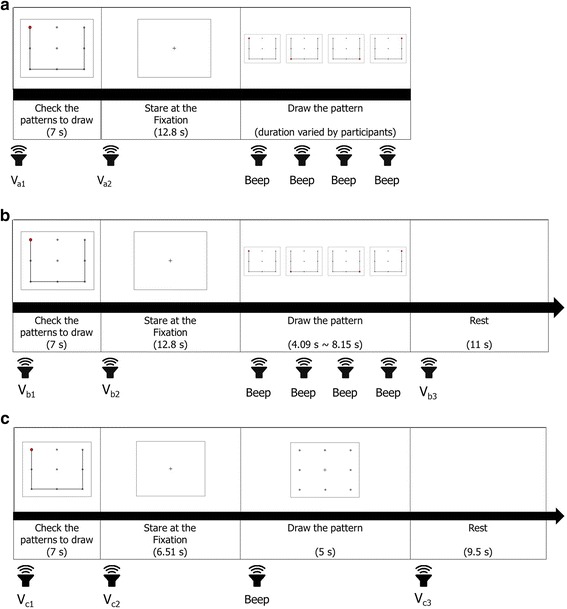
Experimental procedures of the three sessions for participants with ALS. **a** Instruction, **b** practice, **c** exercise. The numbers within the blanks denote the duration of image display. The sound images denote beeps or recorded verbal instructions (noted as V_x_, where x denotes an instruction index). The verbal instructions consisted of cues for participant action. For the full instructions given to the participants, see Appendix

For the experiments with participants with ALS, the experimental procedures were modified to facilitate learning and to minimize user feedback. Each experiment was composed of three sessions: instruction, practice, and exercise (see Fig. 3). During the instruction session, the fundamental concepts of *eye-writing* were explained. First, a pattern corresponding to the number ‘0’ was shown to the participants for 7 s, allowing them to identify the number pattern. The patients were then asked to fix their gaze at the center point in the screen, after which a small red dot was displayed at the starting point. The red dot then disappeared and soon reappeared at the next corner point. In this manner, the red dot sequentially moved and drew the number pattern until reaching the end point. Each participant *eye-wrote* the number pattern by shifting his/her gaze and following the sequential displacement of the red dot. The disappearance and appearance of the red dot were manually controlled by an experimenter; the experimenter changed the location of the red dot when saccadic movement of the participant was observed. When the red dot was displaced, a beep was presented to the participants to notify them of this displacement. In this instruction session, the participants only *eye-wrote* the number ‘0.’ Please note that the purpose of this session was to let the participants know how to *eye-write* a number with their eye-movements. The experimenter could sometimes misrecognize the participants’ eye-movements, but such a little misrecognition did not significantly affect the training of *eye-writing*.

In the practice session, the participants were asked to *eye-write* all numbers from ‘0’ to ‘9.’ The procedure of the practice session was the same as in the instruction session, except that the position of the red dot shifted automatically. The participants were given a short rest of 11 s before *eye-writing* next the number in this session. The exercise session was the same as the practice session, except that the time frame for *eye-writing* a number was fixed to 5 s and no guide line or red dot was displayed (only dots on a 3 × 3 grid were displayed). A 9.5-s short break was given between consecutive eye-writings to prevent eye fatigue of the participants for the exercise session. This session was repeated three times. However, participant No. 19 only performed the session twice because of fatigue. The overall experimental procedures of the three sessions—instruction, practice, and exercise—were verbally explained to the participants (see Appendix for further details).

To avoid fatigue, a relatively long resting period was given to the participants between sessions and trials (a trial denotes *eye-writing* of 0 to 9). The durations of the resting period varied according to the participants’ opinion (varied from 18 s to 11 min).

### Reconstruction of eye movement traces

**Fig. 4:**
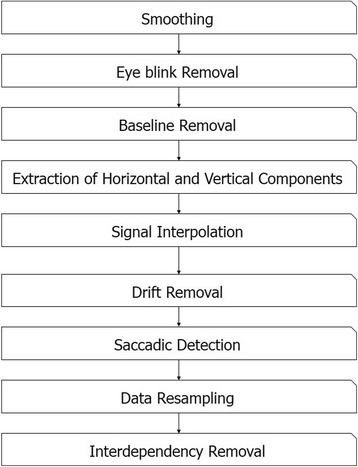
Overall procedure for the reconstruction of eye movement traces

EOG data were obtained during the exercise sessions, and the eye movement traces were reconstructed using signal processing techniques introduced in previous studies [17, 21–23] (see Fig. 4 for the overall procedure). First, source signals were down-sampled at a sampling rate of 64 Hz and median-filtered. High-frequency noises were removed in this step. Second, eye blink artifacts were automatically detected and removed as described in [23]. The time interval, including eye blink artifacts, was determined using the following equation:

1 $$ R=\left\{\left[T\left({Max}_{i-j}\right)-\left|{W}_{f\left(T\left({Max}_{i-j}\right)\right)}\right|,\kern0.5em T\left({Min}_i\right)\right]\right\}, $$

where *f*(*t*) is the output of a digital filter that emphasizes eye blink signals [24], and *T*(*Max*_*i*_) and *T*(*Min*_*i*_) are time points of the *i*th local maximum and minimum, respectively. Third, baselines were removed for each signal channel. A median value calculated from 100 ms of preceding data was used as the baseline of each *eye-written* number. Fourth, the horizontal and vertical EOG components were obtained. The horizontal EOG component was obtained by subtracting the EOG signal of the left channel from that of the right channel, whereas the vertical component was obtained by subtracting the EOG signal acquired at the channel below the eye from the corresponding channel above the eye. The fifth and sixth steps consisted of signal interpolation due to eye blinks and the removal of low-frequency monotonic drifts, respectively. Specifically, missing data due to the eye blink removal process were linearly interpolated using adjacent signals, and the low-frequency drifts were removed using linear regression. Saccadic eye movements were then detected using the *continuous wavelet transform-saccade detection* algorithm introduced by Bulling et al. [22], which extracts signals whose absolute wavelet coefficient values exceed a preset threshold (*θ*). The wavelet coefficient ${C}_b^a$ of data *s* at scale *a* and position *b* is defined as

2 $$ {C}_b^a(s)={\int}_{\mathrm{\mathbb{R}}}s(t)\frac{1}{\sqrt{a}}\overline{\psi \left(\frac{t-b}{a}\right)} dt $$

where *ψ* represents a Haar mother wavelet. The wavelet scale was set to 20, as proposed by Bulling et al. The threshold value was derived by collecting data from an additional participant (male, 24 years old) who did not participate in the main experiments. To derive the threshold value, saccadic regions in the data were manually labeled, and the minimum value of the wavelet coefficient within the saccadic region was used as the threshold. After saccade detection, the signals were resampled to have the same Euclidean distance between adjacent points, and the signal sizes were normalized to make both the width and height to be one. In addition, the interdependency between horizontal and vertical components was removed without any calibration data, as follows:

3 $$ {{\mathrm{EOG}}^c}_v={\mathrm{EOG}}_v\hbox{--} \upalpha\ {\mathrm{EOG}}_h, $$

where EOG^*c*^_*v*_ is the compensated vertical component, α is a parameter that describes the amount of interdependency between the horizontal and vertical components, and EOG_*v*_ and EOG_*h*_ are the vertical and horizontal EOG components, respectively.

Unlike the conventional method that requires some preliminary calibration data [23], we developed a new method for estimating α without any calibration. Data in a sequence were differentiated by a finite difference function:

4 $$ {\mathrm{s}}^{\prime }(t)=s\left(t+1\right)\hbox{--} s\left(t-1\right). $$

**Fig. 5:**
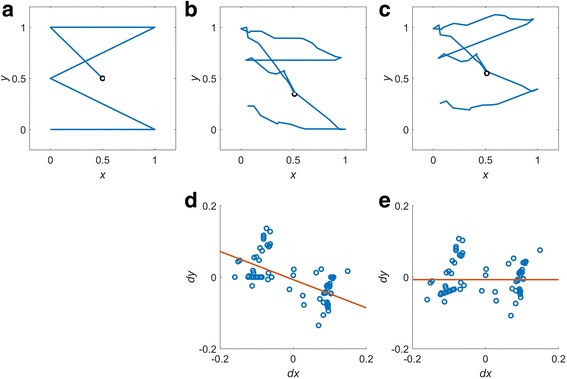
Removal of interdependency between horizontal and vertical components. **a** Reference pattern, **b** signal before interdependency removal, **c** signal after interdependency removal, **d** difference plot of (**b**), and **e** difference plot of (**c**). *x* and *y* are the the normalized values of the horizontal and vertical signal, respectively; and *dx* and *dy* are the difference values of x and y, respectively

The value of α can be estimated even in the absence of calibration data, simply by linearly regressing the values in the differentiated data. The processes for estimating α and removing the interdependency are described in Fig. 5. Figure 5b shows an example of an *eye-written* character ‘3,’ where the *eye-written* shape is severely skewed compared with the template pattern (Fig. 5a). We found that the degree of skewness can be well described as the linear regression slope of data distribution in the finite difference space of adjacent values (Fig. 5d). The parameter α was determined as the value that makes the slope to be zero, as shown in Fig. 5e. Using the determined α value, the *eye-written* pattern became more similar to the template pattern (Fig. 5c).

### Classifiers

We tested four different types of classifiers for the proposed system: dynamic time warping (DTW), dynamic positional warping (DPW), a combination of DTW with support vector machine (SVM), and a combination of DPW with SVM. DTW measures dissimilarity between two time-series with different lengths and is often utilized for handwritten character recognition or signature verification [25, 26]. DPW is an extended version of DTW that has been specialized for two-dimensional shapes by allowing warping on value axes [27]. Conventional DTW/DPW have utilized a very simple classifier which just choose one with the minimum distance from the reference. In this study, we tried to verify whether the combination of the DTW/DPW with a more advanced classifier could increase recognition accuracy. In this study, SVM was chosen as it is a well-known classifier that is used in many research fields [22, 28–30].

Since it is not yet possible to use the dissimilarities obtained from DTW or DPW as features for SVM, we introduced a new way of combining the DTW/DPW and SVM.

In DTW, the dissimilarities between two signals (*A* and *B*) are defined as follows:

5 $$ {d}_{dtw}=\uptau \left({L}_A,{L}_B\right) $$

6 $$ \uptau \left(i,j\right)=\left|\left\{A(i)-A\left({i}_{prev}\right)\right\}-\left\{B(j)-B\left(j-{j}_{prev}\right)\right\}\right|+\upomega \left(i,j\right) $$

7 $$ \upomega \left(i,j\right)=\underset{c}{\min}\left\{\uptau \left(i-{C}_A(c),j-{C}_B(c)\right)\right\}, $$

where *L*_*A*_ and *L*_*B*_ are the lengths of two signals, *i*_*prev*_ = *i* − 1, *j*_*prev*_ = *j* − 1, τ(1, 1) = 0, and *C*_*A*_ and *C*_*B*_ are the pairs of constraints for warping time. The constraints (*C*_*A* , *B*_) are defined as

8 $$ {C}_{A,B}=\left\{\left(1,m\right),\left(m,1\right)|1\le m\le M\right\}, $$

where *M* is the maximum distance for time-warping.

In DPW, the definition of dissimilarity is similar to that in DTW. Specifically, dissimilarity can be written by substituting *i*_*prev*_ and *j*_*prev*_, as follows:

9 $$ {i}_{prev}=i-{C}_A\left({c}_{\mathrm{min}}\left(i,j\right)\right) $$

10 $$ {j}_{prev}=j-{C}_B\left({c}_{min}\left(i,j\right)\right) $$

11 $$ {c}_{min}\left(i,j\right)=\underset{c}{\mathrm{argmin}}\left\{\uptau \left(i-{C}_A(c),j-{C}_B(c)\right)\right\}. $$

Detailed descriptions of DTW and DPW can be found in [23, 27].

To combine DTW/DPW with SVM, we used a set of normalized dissimilarities to the templates as a feature vector for SVM. Dissimilarities to all templates were calculated for a test signal using DTW/DPW. These dissimilarities were then divided by the normalization factors of each template. These normalization factors were calculated using training data. Specifically, dissimilarities to the corresponding template were calculated for each of the training datasets, and the mean dissimilarity in a class was utilized as the normalization factor of the class [31].

### Validation

We evaluated the performance of each method by leave-one-subject-out testing to verify whether our *eye-writing* approach is user-independent. Each participant’s data were tested individually, where data from all healthy participants except the participant being tested were used for training. Data from participants with ALS were not used to train the models due to the limited sample size. All the data during the exercise sessions were used for the validation. Please note that three sets of *eye-written* numbers were collected per participant during the exercise session, except the participant #19.

Recognition performance was evaluated in three different ways to express different aspects of the results. First, the overall recognition performance was measured as follows:

12 $$ {\mathrm{ACC}}_{overall}={\mathrm{TP}}_{overall}/{\mathrm{P}}_{overall}, $$

where TP_*overall*_ denotes the number of patterns (*eye-written* numbers) that were correctly classified, and P_*overall*_ is the total number of patterns. The overall performance was measured for each group (healthy or ALS) to enable comparison of the performance of participants with ALS versus that of healthy participants. Second, the recognition performance were evaluated for each participant as follows:

13 $$ {\mathrm{ACC}}_{particpant}={\mathrm{TP}}_{participant}/{\mathrm{P}}_{participant}, $$

where TP_*participant*_ denotes the number of the patterns that were correctly classified for a given participant, and P_*participant*_ is the total number of patterns attempted by the participant. The performance was also evaluated for each of the Arabic numbers, as follows:

14 $$ {\mathrm{Precision}}_{digit}=\raisebox{1ex}{${\mathrm{TP}}_{digit}$}\!\left/ \!\raisebox{-1ex}{$\left({\mathrm{TP}}_{digit}+{\mathrm{FP}}_{digit}\right)$}\right., $$

15 $$ {Sensitivity}_{digit}\  or\ {\mathrm{Recall}}_{digit}=\raisebox{1ex}{${\mathrm{TP}}_{digit}$}\!\left/ \!\raisebox{-1ex}{$\left({\mathrm{TP}}_{digit}+{\mathrm{FN}}_{digit}\right)$}\right., $$

16 $$ \mathrm{F}1\_{\mathrm{score}}_{digit}=\frac{2{\mathrm{Precision}}_{digit}\bullet {\mathrm{Recall}}_{digit}}{{\mathrm{Precision}}_{digit}+{\mathrm{Recall}}_{digit}}, $$

where TP, FP, and FN represent the true positive, false positive, and false negative rates, respectively.

## Results

The overall accuracies of the proposed method with data from healthy participants were 92.41% 94.07%, 94.07%, and 95.37% for DTW, DTW + SVM, DPW, and DPW + SVM, respectively. We found that the recognition rates of EOG-based *eye-writing* could exceed 95%, demonstrating its viability as an alternative to camera-based eye tracking interfaces. The DPW + SVM combination resulted in the highest overall recognition rate in healthy participants. DPW showed better performance than DTW; moreover, the combination with SVM effectively increased the overall recognition rates for both dissimilarity measures. The recognition rates dropped drastically when the same method was applied to participants with ALS: 83.75%, 81.25%, 87.50%, and 85.00% for DTW, DTW + SVM, DPW, and DPW + SVM, respectively. Specifically, the best recognition rate was 87.50% when DPW alone was used. Despite this drop, this rate was still higher than that reported in a previous study, where the recognition rate of binary classification was just 71% for an individual with ALS [9].

Table 1 shows the recognition rates of the proposed method for each participant. The DPW + SVM combination showed the best mean recognition rate (94.37%); other classifiers resulted in slightly lower recognition rates (93.10% for DPW, 92.38% for DTW + SVM, and 91.27% for DTW). However, no statistically significant differences were found among the performances of these classifiers. It is notable that one patient with ALS (participant no. 20) showed the lowest recognition rate of all participants, while the recognition rates of the other two participants with ALS were comparable to those of healthy participants.

**Table 1:** Recognition accuracies for each participant with different classifiers

Participant Status	Participant number	DPW + SVM	DPW	DTW + SVM	DTW
Healthy	1	96.67	93.33	96.67	96.67
	2	83.33	90.00	83.33	83.33
	3	90.00	100.00	86.67	93.33
	4	96.67	93.33	86.67	96.67
	5	100.00	100.00	100.00	100.00
	6	100.00	100.00	100.00	93.33
	7	100.00	96.67	90.00	86.67
	8	100.00	93.33	100.00	96.67
	9	100.00	96.67	96.67	96.67
	10	93.33	96.67	96.67	96.67
	11	96.67	93.33	96.67	96.67
	12	96.67	90.00	100.00	93.33
	13	96.67	93.33	90.00	93.33
	14	96.67	90.00	93.33	86.67
	15	100.00	100.00	96.67	100.00
	16	96.67	90.00	90.00	83.33
	17	93.33	90.00	96.67	80.00
	18	86.67	86.67	93.33	90.00
	Avg.	95.74	94.07	94.08	92.41
	Std.	4.83	4.21	5.18	6.03
ALS	19	95.00	85.00	90.00	90.00
	20	70.00	80.00	66.67	73.33
	21	93.33	96.67	90.00	90.00
	Avg.	86.11	87.22	82.22	84.44
	Std.	13.98	8.55	13.47	9.62
Overall	Avg.	94.37	93.10	92.38	91.27
	Std.	7.16	5.33	7.68	6.95

## Discussion

**Fig. 6:**
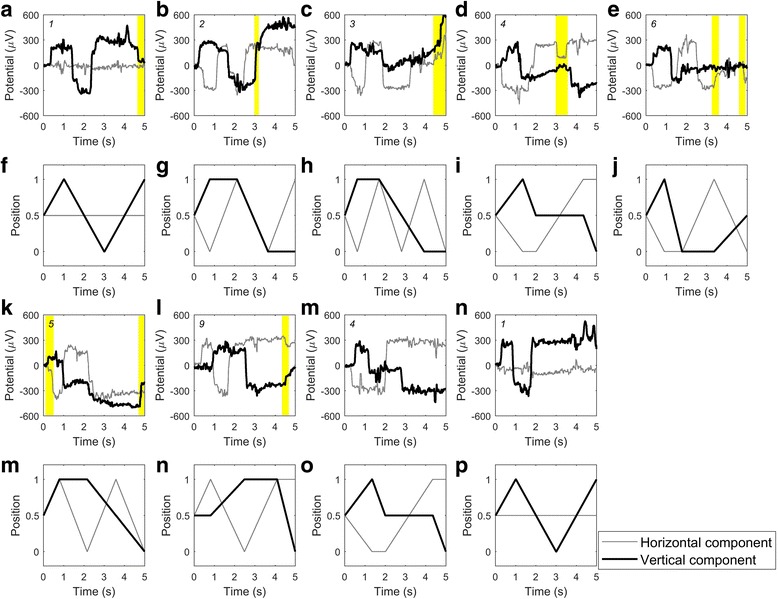
Misrecognized signals of a participant with ALS. The first and third rows show the misrecognized signals of participant No. 20. The second and fourth rows show their corresponding templates. The numbers on the top left corner of each axis denote the intended numbers to be written by the participant. Unexpected eye movements (additional or missing saccades) were highlighted by visual inspection of the signal. All preprocessing procedures were minimized (baseline removal and median filtering only) for visual inspection. The template patterns did not include eye fixation parts. First seven errors (i.e., 7 pairs of panels, **a**-**f**, **b**-**g**, **c**-**h**, **d**-**i**, **e**-**j**, **k**-**m**, and **l**-**n**) were associated with additional or missing saccades. The other two errors (**m**-**o** and **n**-**p** pairs) were caused by high frequency ripples possibly originating from microsaccades

The main aim of the present study was to prove that EOG-based *eye-writing* is a feasible human computer interface (HCI) tool for the individuals with amyotrophic lateral sclerosis (ALS). The promise of the EOG-based *eye-writing* was demonstrated by a number of previous studies [17, 18, 32], but it has not yet been tested on individuals with ALS, to the best of our knowledge. Results summarized in Table 1 showed that the recognition accuracies for two out of three participants with ALS (nos. 19 and 21) were as high as the healthy participants, but the recognition rate was relatively lower in participant no. 20. In an attempt to better understand why participant no. 20 exhibited such low recognition rate, we analyzed the participant’s raw signals. This analysis showed that many of the signals were misrecognized because they included additional or missing saccades. Figure 6 shows nine misidentified raw EOG signals that were recorded while the participant was *eye-writing* specific patterns. As shown in Fig. 6, seven of the nine errors were associated with additional or missing saccades. However, this type of error decreased significantly as the trials proceeded. Specifically, five miswritten numbers were observed in the first trial, two in the second trial, and none in the last trial. This finding indicates that, while the participant had difficulty using the system initially, he quickly adapted to it. It would be an important topic to improve the usability of the system in order for the users to get accustomed to the proposed system more easily. The other two errors were caused by high-frequency ripples in the signal, which possibly originated from microsaccades. We predict that these errors could be further reduced by developing new features that consider the global shapes of the numbers. Redesigning the number shape may be another option for increasing the recognition rates.

The small number of the participants with ALS may not be enough to prove the feasibility of the *eye-writing* system in clinical applications; however, we would like to emphasize that this was the first study that applied the *eye-writing* technology to the participants with ALS. Although EOG-based HCI has been studied by many research groups with the ultimate goal for assisting the people with ALS [17, 33–39], most of the previous studies evaluated their systems using data acquired from healthy participants. To the best of our knowledge, there has been only a single study that tested the developed system to individuals with ALS [9], where only one participant with ALS was recruited to evaluate an EOG-based HCI system for binary communication (mean accuracy of the binary classification was just 71%). We believe that the results of the current study are very promising as two out of three participants with ALS showed the overall recognition rates higher than 93%. Nevertheless, application of our system to more numbers of participants with ALS and evaluation of the recognition rates with respect to the symptom severity scores (ALSFRS) would be important topics that we would like to pursue in future studies. We believe that development of a mobile application incorporated with a mobile EOG recording device would help to recruit more participants with ALS.

Another issue to be addressed for implementing practical EOG-based eye-computer interface would be the wavelet threshold (*θ*) introduced in Reconstruction of eye movement traces section This threshold affects the sensitivity of detecting saccadic movements. When the threshold becomes higher, lower frequency noises are removed together with low-speed eye-movements. An optimal threshold may minimize the loss of the eye-movement information and remove the noises as much as possible. In our experiments, the threshold was derived from data of a single participant. To verify the stability of this approach, we derived the threshold values from all the individual participants and tested how much the changes in the threshold values influence the performance of recognizing *eye-written* numbers.

**Fig. 7:**
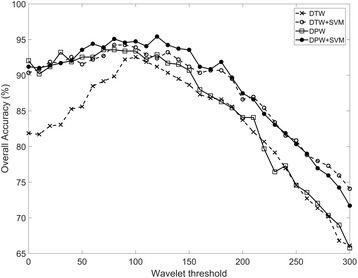
Overall accuracies with respect to wavelet threshold

The derived threshold values for healthy participants were 148.98 ± 64.80, ranging from 52.08 to 278.82. The values derived from the participants with ALS were 200.55, 256.56, and 117.33 for participants #19, #20, and #21, respectively. They were all within the range of the healthy participants. The changes in overall accuracy (*Acc*_*overall*_) are depicted in Fig. 7. In this experiment, SVM significantly increased the accuracy when it was combined with DTW or DPW (*p* < 0.001, Wilcoxon’s signed rank test with Bonferroni correction). The best recognition rate (95.42%) was achieved with the threshold value of 120 when the combination of DPW and SVM was used. The recognition rates of all the classifiers decreased as the threshold increases after achieving their bests in between 70 and 120. This trend occurred because higher wavelet threshold removed the details of signals [40] (shapes of the *eye-written* characters in this study). It is noteworthy that the combination of SVM with DPW/DTW showed enhanced stability of the recognition rate with respect to the varying wavelet thresholds. Since recognition rates varies from 70 to 95% according to the wavelet parameter, this would be one of the important advantage of combining SVM with DPW/DTW. Moreover, SVM also has a big potential to further increase the recognition rates if increased size of training data are used.

One of the disadvantages of SVM would be that it requires additional time for training procedure. We measured the time durations of training and test procedures of each classifier to validate their usability for practical applications. They were tested using Matlab 2015b on Windows 7 (Intel i5–2320 CPU and 16GB RAM). In our experiment, SVM with DTW classifier spent 79.152 s for training 17 subjects’ data, and SVM with DPW classifiers spent 99.661 s on average. This time, however, does not affect its usability for real time applications. The testing time, which is important for a practical use, were 62 ms, 116 ms, 118 ms, and 189 ms, for DTW, DTW + SVM, DPW, and DPW + SVM, respectively. It is clear that DTW/DPW run faster without SVM, but the combined approach was still applicable for real time applications, as we can still recognize five numbers per second.

In this study, we developed a series of computational methods to more accurately recognize 10 Arabic numbers. One of the important methods that we developed in this study was one for removing interdependency between horizontal and vertical eye-movement components in EOG. Conventional method [22] needed some preliminary calibration data to determine a parameter describing the amount of interdependency between the horizontal and vertical components, whereas the proposed algorithm does not need any calibration to determine the same parameter. The other method proposed in this study was one to combine DTW or DPW with SVM, which has not been introduced before this work. The combination of DPW with SVM resulted in the highest overall recognition rates, which was higher than those of the conventional DTW and DPW algorithms.

We also proposed a training procedure to efficiently explain to the user, with minimal user feedback, how to *eye-write*. This approach is particularly of importance in practical applications of the proposed *eye-writing* system to patients with ALS because it is generally difficult to receive immediate and accurate responses or feedbacks from these individuals.

The recognition rates can be affected by the shape of the Arabic numbers. Table 2 shows the classification accuracies for each Arabic number. In calculating the mean classification accuracies, data from all the participants were included except the participant no. 20 (an individual with ALS who showed lowest recognition rates). The data were excluded so as to avoid potential bias in the results. As seen from the table, most of the errors occurred during the writing of two numbers, ‘4’ and ‘7.’ (Mean accuracies without the two numbers are 96.79%, 95.33%, 95.58%, and 94.74% for DPW + SVM, DPW, DTW + SVM, and DTW, respectively) Specifically, the F1 scores of these two numbers were lowest for all classifiers except DPW. These results suggest that changes in the shape of the two numbers, 4 and 7, might enhance the overall recognition rates.

**Table 2:** Recognition rates of each Arabic number with different classifiers

Number	DPW + SVM			DPW			DTW + SVM			DTW		
	PR	SE/RE	F1	PR	SE/RE	F1	PR	SE/RE	F1	PR	SE/RE	F1
0	96.72	100.00	98.33	98.15	89.83	93.81	93.10	91.53	92.31	96.36	89.83	92.98
1	86.15	94.92	90.32	96.30	88.14	92.04	95.08	98.31	96.67	96.61	96.61	96.61
2	98.28	96.61	97.44	98.33	100.00	99.16	94.83	93.22	94.02	98.21	93.22	95.65
3	96.67	98.31	97.48	100.00	96.61	98.28	95.08	98.31	96.67	96.61	96.61	96.61
4	98.00	83.05	89.91	81.82	91.53	86.40	87.72	84.75	86.21	77.05	79.66	78.33
5	100.00	98.31	99.15	100.00	96.61	98.28	98.31	98.31	98.31	98.28	96.61	97.44
6	95.16	100.00	97.52	91.67	93.22	92.44	92.19	100.00	95.93	83.10	100.00	90.77
7	89.47	86.44	87.93	85.07	96.61	90.48	90.57	81.36	85.71	87.72	84.75	86.21
8	98.33	100.00	99.16	96.72	100.00	98.33	95.08	98.31	96.67	96.61	96.61	96.61
9	94.92	94.92	94.92	94.44	86.44	90.27	94.83	93.22	94.02	94.55	88.14	91.23
Avg.	95.37	95.25	95.22	94.25	93.90	93.95	93.68	93.73	93.65	92.51	92.20	92.24
St dev.	4.33	5.91	4.23	6.27	4.81	4.38	2.94	6.34	4.41	7.35	6.45	6.05

PR, SE, RE, and F1 denote precision, sensitivity, recall, and F1 score, respectively. The recognition rates were computed from the data of all the participants except participant #20

## Conclusion

Here we demonstrated that EOG-based *eye-writing* can be an alternative communication tool for individuals with ALS. To implement this practical communication tool, we designed simplified patterns of Arabic numbers and proposed a series of algorithms for efficiently reconstructing and identifying the eye movement traces. Through our experiments with 18 healthy participants and three participants with ALS, we confirmed that our EOG-based *eye-writing* system can be successfully used as a communication tool by individuals with ALS. Although performance was lower in participants with ALS than in healthy participants, their performance could potentially be improved by consistent practice. Moreover, our proposed system could increase the usability of this technique if it is applied to portable hardware.

## Appendix

### Verbal instructions for participants with ALS

This section lists the verbal instructions during data acquisition for participants with ALS. The item numbers (V_x_) are the same as in Fig. 4.

### Instruction session

- V_a1_: Open your eyes and look at the number to draw.
- V_a2_: Stare at the fixation mark. Draw the number when you hear the beep. To draw the number, follow the red dot that shifts position with the beep.
- V_a3_: Close your eyes and take a rest.

### Practice session

- V_b1_: Open your eyes and look at the number to draw.
- V_b2_: Stare at the fixation mark. Draw the number when you hear the beep. To draw the number, follow the red dot that shifts with the beep.
- V_b3_: Close your eyes and take a rest.

### Exercise session

- V_c1_: Open your eyes and look at the number to draw.
- V_c2_: Stare at the fixation mark. Draw the number when you hear the beep.
- V_c3_: Close your eyes and take a rest.

## Abbreviations

ALS: Amyotrophic lateral sclerosis
ALSFRS: ALS functional rating scale
DPW: Dynamic positional warping
DTW: Dynamic time warping
EOG: Electrooculogram
HCI: Human-computer interface
IRB: Institutional review board
SVM: Support vector machine

## References

[1] Beukelman D, Fager S, Nordness A. Communication support for people with ALS. Neurol Res Int. 2011;2011:6. https://www.hindawi.com/journals/nri/2011/714693/. 10.1155/2011/714693 PMC3096454 21603029

[2] Communication Guide. ALS Assoc. 2016. Available from: http://www.alsa.org/als-care/augmentative-communication/communication-guide.html

[3] Murphy J (2004). Communication strategies of people with ALS and their partners. Amyotroph. Lateral Scler. Other Motor Neuron Disord.

[4] Calvo A, Chiò A, Castellina E, Corno F, Farinetti L, Ghiglione P, et al. Eye tracking impact on quality-of-life of ALS patients. Lect Notes Comput Sci. 2008;5105:70–7.

[5] Hwang C-S, Weng H-H, Wang L-F, Tsai C-H, Chang H-T (2014). An eye-tracking assistive device improves the quality of life for ALS patients and reduces the caregivers’ burden. J Mot Behav.

[6] Caligari M, Godi M, Guglielmetti S, Franchignoni F, Nardone A (2013). Eye tracking communication devices in amyotrophic lateral sclerosis: impact on disability and quality of life. Amyotroph lateral Scler Front Degener.

[7] Morimoto CH, Mimica MRM (2005). Eye gaze tracking techniques for interactive applications. Comput Vis Image Underst.

[8] Holmqvist K, Nyström M, Mulvey F (2012). Eye tracker data quality: what it is and how to measure it. Proc Symp eye Track Res Appl.

[9] Kaethner I, Kuebler A, Halder S, Kathner I, Kubler A, Halder S (2015). Comparison of eye tracking, electrooculography and an auditory brain-computer interface for binary communication: a case study with a participant in the locked-in state. J Neuroeng Rehabil J NeuroEng Rehab.

[10] Bulling A (2010). Eye Movement Analysis for Context Inference and Cognitive-awareness: Wearable Sensing and Activity Recognition Using Electrooculography. ETH Zurich.

[11] Barea R, Boquete L, Mazo M, López E (2002). Wheelchair guidance strategies using EOG. J Intell Robot Syst Theory Appl.

[12] Wijesoma WS, Wee KS, Wee OC, Balasuriya AP, San KT, Soon KK (2005). EOG based control of mobile assistive platforms for the severely disabled.

[13] LaCourse JR, Hludik FCJ (1990). An eye movement communication-control system for the disabled. IEEE Trans Biomed Eng.

[14] Kim MR, Yoon G (2013). Control signal from EOG analysis and its application. Int J Electr Comput Electron Commun Eng.

[15] Kaufman AE, Bandopadhay A, Shaviv BD. An Eye Tracking Computer User Interface. San Jose: IEEE Symp Res Front Virtual Real; 1993. p. 120–1.

[16] Yan M, Tamura H, Tanno K. A study on gaze estimation system using cross-channels electrooculogram signals. Int Multiconf Eng Comput. 2014;1:112–6.

[17] Tsai J-Z, Lee C-K, Wu C-M, Wu J-J, Kao K-P (2008). A feasibility study of an eye-writing system based on electro-oculography. J Med Biol Eng.

[18] Lee K, Chang W, Kim S, Im C (2016). Real-time “eye-writing” recognition using electrooculogram (EOG). Trans Neural Syst Rehabil Eng.

[19] Ohki M, Kanayama R, Nakamura T, Okuyama T, Kimura Y, Koike Y (1994). Ocular abnormalities in amyotrophic lateral sclerosis. Acta Otolaryngol Suppl.

[20] Cedarbaum JM, Stambler N, Malta E, Fuller C, Hilt D, Thurmond B (1999). The ALSFRS-R: A revised ALS functional rating scale that incorporates assessments of respiratory function. J Neurol Sci.

[21] Joyce CA, Gorodnitsky IF, King JW, Kutas M (2002). Tracking eye fixations with electroocular and electroencephalographic recordings. Psychophysiology.

[22] Bulling A, Ward JA, Gellersen H, Tröster G (2011). Eye movement analysis for activity recognition using electrooculography. IEEE Trans Pattern Anal Mach Intell.

[23] Chang W-D, Cha H-S, Im C-H. Removing the interdependency between horizontal and vertical eye-movement components in electrooculograms. Sensors (Switzerland). 2016;16(2). Article ID: 227. 10.3390/s16020227 PMC4801603 26907271

[24] Chang W-D, Cha H-S, Kim K, Im C-H (2015). Detection of eye blink artifacts from single prefrontal channel electroencephalogram. Comput Methods Prog Biomed..

[25] Feng H, Wah CC (2003). Online signature verification using a new extreme points warping technique. Pattern Recogn Lett.

[26] Shin J (2004). On-line cursive hangul recognition that uses DP matching to detect key segmentation points. Pattern Recogn.

[27] Chang W-D, Shin J (2009). Dynamic positional warping: dynamic time warping for online handwriting. Int J Pattern Recognit Artif Intell.

[28] Hodge VJ, Austin JIM. A Survey of Outlier Detection Methodologies. Artif Intell Rev. 2004;22(2):85–126.

[29] Markou M, Singh S (2003). Novelty detection: a review—part 2: neural network based approaches. Signal Process.

[30] Li S, Zhou W, Yuan Q, Geng S, Cai D (2013). Feature extraction and recognition of ictal EEG using EMD and SVM. Comput Biol Med.

[31] Chang W-DCW-D, JSJ S (2008). DPW Approach for Random Forgery Problem in Online Handwritten Signature Verification.

[32] Chanceaux M, Rynik V, Lorenceau J, Syst L (2014). Writer recognition in cursive eye writing : A Bayesian model BAP-EOL Model.

[33] Tomita Y, Igarashi Y, Honda S, Matsuo N (1996). Electro-Oculography Mouse for Amyotrophic Lateral Sclerosis Patients.

[34] Yamagishi K, Hori J, Miyakawa M (2006). Development of EOG-based communication system controlled by eight-directional eye movements.

[35] Hori J, Sakano K, Saitoh Y (2004). Development of communication supporting device controlled by eye movements and voluntary eye blink.

[36] Lee JS, Lim GG, Kwon SJ, Park KS (2015). Non-contact blink detection glasses utilising transparent conductive film for binary communication. Electron Lett.

[37] Yan M, Go S, Tamura H (2014). Communication system using EOG for persons with disabilities and its judgment by EEG. Artif Life Robot.

[38] Yagi T, Kuno Y, Koga K, Mukai T (2007). Drifting and blinking compensation in electro-oculography (EOG) eye-gaze interface.

[39] Tsai J-Z, Chen T-S (2009). Eye-writing communication for patients with amyotrophic lateral sclerosis.

[40] Stéphane M. A Wavelet Tour of Signal Processing. Burlington: Academic Press; 2009.

